# Bridging or Bonding: An Organizational Framework for Studying Social Capital in Kindergartens

**DOI:** 10.3390/ijerph18052663

**Published:** 2021-03-06

**Authors:** Berit Irene Vannebo, Elin Birgitte Ljunggren

**Affiliations:** 1 Faculty of Social Sciences, Nord University, 7600 Levanger, Norway; 2 Section of Social Science, Queen Maud University College, 7044 Trondheim, Norway; elin.b.ljunggren@dmmh.no

**Keywords:** social capital, ECEC, organizations, community, qualitative study

## Abstract

The article develops our understanding of social capital by analyzing social capital as an organizational phenomenon. The analysis is based on qualitative data consisting of interviews and documents obtained from six different kindergartens in Norway. Kindergartens are used as a “prism” through which we can understand how social capital is formed—and the mechanisms that shape the development of various forms of networks within welfare organizations. More specifically we look at drop-in kindergartens. The specific purpose of these kindergartens is to provide open and inclusive arenas that promote integration and community. We find that the kindergartens vary in the degree to which they succeed in building bridging forms of networks and communities. Using concepts from organizational theory and Wenger’s (1998) theory of communities of practice, we find that formal organizational factors such as ownership, organizational goals, profiling, location, and educational content impact the formation of bridging forms of social capital. The composition of the user groups and the user groups’ motivation for participating most clearly affect the conditions for community formation. The composition of the user groups is the result of a number of organizational factors and organizational mechanisms. Kindergartens that have a heterogeneous user group, and a user group with a community orientation (Morse 2006), are more successful at creating bridging types of social networks.

## 1. Introduction

### The Kindergarten as a Tool for Social and Political Change 

Social inequality is a growing problem in Norway, and research has shown that marginalization of certain groups starts early. Equalization of inequality is a political goal, and early intervention is a tool for producing social equalization in Norway [1]. An important task for achieving the goal of social equalization is to build institutions that can give marginalized groups access to social networks and access to arenas with important resources that promote inclusion [2]. Social inclusion and social equalization thus become shared political goals, and building inclusive communities is on the political agenda. In this political project, the kindergarten becomes a policy tool utilized by the Norwegian welfare state in their work to create more just and equal opportunities and to level social differences. In this article, we are interested in understanding how kindergartens contribute to social inclusion by facilitating the formation of various forms of communities, and thereby producing various forms of social capital.

Social capital is indicative of the amount, and quality, of the social relations and networks in a society [3]. Social capital can thus be understood as a societal good, and as a measure of inclusion and participation in a society. Research on social capital in Norway has been concerned with looking at the connections between networks and political and local participation [4]. In this paper, we propose an alternative perspective focusing on how social capital is built and formed—and on the mechanisms that are involved in the building of different forms of social capital inside organizations with social and political goals. We look at these organizations as unique community arenas, and analyze the organizational frameworks that facilitate the building of social capital inside organizations. Social capital can be produced or deteriorate, and several key organizational features determine which processes unfold. We can thus see organizations as sociopolitical tools that can both facilitate and hinder the production of social capital.

Social capital is a concept that can be used analytically to study a range of phenomena, but so far it has not been used to study kindergartens as organizations. The connection between social capital and a strong and democratic civic society has been a focus of many studies [5,6,7]. Several have also researched the linkages between organizational membership and social capital, looking especially at connections between voluntary organizations and local networks [4,8]. We know less about what organizations do to build social capital, and about how organizations such as kindergartens can facilitate the building of various forms of social capital. In this article, we focus on the organizational features—the formal and informal structures—that impact how kindergartens organize the building of communities and networks. We look at which organizational features are crucial for the building of social capital in kindergartens, and which ensure that kindergartens become open and communal arenas. By focusing on how social capital is produced in kindergartens, this article contributes to research on the importance of social capital for combating social inequality, and to research on the political and social functions that kindergartens serve and their role in creating inclusive communities.

Several have written on the importance of cross-sectorial collaborations in strengthening and supporting early intervention among marginalized children and families [9,10]. There is, however, less research on drop-in kindergartens as arenas for integration and inclusion [11,12]. Drop-in kindergartens are particularly suited as cases to examine. These kindergartens provide us with a “prism” by which to study the connection between the building of social capital and attaining political welfare goals as they 1) have as a goal to contribute to building networks among minorities in—and include minorities in—Norwegian society, 2) rely on cross-sectorial collaborations between different agencies and collaboration between the public and the voluntary sector, and 3) mirror a development towards more “customer-oriented” welfare services and towards the building of open public arenas for meeting and building new forms of community. We thus chose to look at how these kindergartens—which are defined as open and inclusive forms of community—facilitate the building of different forms of social capital. Our research questions in this article are as follows:

*Which kinds of communities are built in drop-in kindergartens? How do differences in ways of organizing kindergartens impact which forms of social capital can be built in these organizations?*

## 2. Theory 

### 2.1. Social Capital as a Resource

Research on social capital in Norway has focused on the importance of social networks for minority groups’ inclusion and participation in Norwegian society [2,4,13,14]. At the start of 2020, immigrants constituted 14,7 percent of the Norwegian population [15]. The government emphasizes the importance of including and integrating the immigrant population. Social capital is especially important as a resource for including minorities, and ensuring that minorities participate, in local communities and organizations [7,8]. Research has also shown that a strong civic society is important, and that a society in which a large amount of people are engaged in voluntary or organizational networks facilitates the building of new communities where people can experience a sense of belonging and organizations built on principles of participatory democracy [16,17,18]. Research has also been concerned with a third form of social capital—linking social capital—which describes networks as relations of trust between individuals and institutional actors [19]. These perspectives focus on the role of the public in building social capital, on networks that are initiated by public agencies and institutions, or on areas were public institutions are partaking in establishing networks [7,20]. It is becoming increasingly important to design new solutions where residents themselves participate in co-creation and the building of care-oriented communities to complement traditional welfare services [21]. By ensuring that organizations are able to maintain and build social capital among residents, a local community can be developed and strengthened, and one can prioritize measures and activities that attend to the needs of the community. Organizations can, in this way, become tools for policy and produce societal benefits.

Different groups participate in a range of networks where they share a set of common interests or values. These networks constitute a resource, a form of capital, as they give access to goods (material, social, and cultural) that participants in the networks can benefit from. Social networks are referred to as capital because they provide access to resources—through networks we can access information, influence or power, and new connections. Reproduction of inequality, and the concept of social capital, are thus linked to ideas about the investment in and accumulation of goods [22]. Access to capital is inherently linked to equalization of social inequality. By including individuals in networks, they can get access to more resources and goods, which can contribute to social mobility and social equalization. Social capital can, however, also be destructive, and is thus not always a resource for mobility [3].

Putnam distinguishes between two forms of social capital—bonding and bridging social capital [6]. Putnam describes bonding social capital as networks that are close-knit, continuous, and strong. These networks are comprised of individuals with similar backgrounds; they are often homogeneous and resemble a community of like-minded people. These networks can thus also seem excluding by only including those who share similar characteristics. Bridging social capital are networks that are looser and weaker, and they often comprise individuals who belong to different networks, and individuals who have different backgrounds and different cultural, social, and economic resources. Bridging types of networks bring together individuals from various networks and individuals with various characteristics, and they are thus often more inclusive. All forms of social capital are important as resources—for individuals and for societies. However, Putnam is mainly concerned with bridging forms of networks and social capital, as these enable the creation of new forms of community and provide individuals from marginalized communities with access to a broader spectrum of resources than those available in bonding forms of networks. Access to social capital can be linked to opportunities in different arenas, such as education and the labor market, and can also impact different groups’ access to health-promoting initiatives [3,23]. Ideally, welfare and political organizations—such as drop-in kindergartens—should facilitate the building of bridging forms of social capital and networks.

Access to different kinds of networks is often not equally distributed between minorities and the majority. Individuals who have little social capital also often experience having less of other forms of capital, at the same time as individuals with low education or low income experience difficulties in accessing networks with important resources. Groups that are marginalized also often experience that it is easier to gain access to bonding forms of networks where they connect with individuals that are similar to them, and their networks are more often homogenous. Individuals from minorities also often participate in networks where participants have in common that they experience a lack of resources, stigmatization, and exclusion [24]. Several have argued that access to bridging forms of social capital is critical to combat social inequality, and to stimulate social mobility [23,24]. Coleman was especially concerned with the connection between social networks and educational performance. According to Coleman, the key to educational performance is access to local communities with continuous networks that provide access to critical resources. Bonding networks thus have a tendency to reproduce inequality by limiting access to new networks that provide access to new arenas and possibilities for social mobility. Bonding networks thus often become a “strategy for survival, without much impact on individuals’ or groups’ situation” [4] (our translation). It is thus important to look at the composition of user groups in an organization—such as kindergartens—to understand the conditions that facilitate, or hinder, the building of bridging forms of networks and communities.

There is a connection between social capital and community. Community we here see as relatively stable relations between individuals who in various ways meet and interact [25]. Community comprises different networks of individuals who bring with them a range of resources, and different communities provide access to different forms of social capital. Community thus forms the basis for the building of social capital, and social capital is accumulated and activated in communities.

Similarly, different forms of social capital facilitate the building of different forms of community. Bonding social capital facilitates the building of what we here call “bonding communities,” and bridging social capital facilitates the building of “bridging communities.” Bonding and bridging communities are characterized by different forms of activated social capital. Bridging communities can be understood as inclusive communities—communities that bring together individuals with different resources and that provide room for diversity. Bridging communities are characterized by individuals with different social and cultural backgrounds; they are characterized by heterogeneity. Bonding communities are characterized by participants with homogenous characteristics and resources. As bridging and bonding communities provide access to different kinds, and amounts, of resources, they also create different conditions for social mobility. Theoretically, we can expect that communities characterized by heterogeneity—to a greater extent than communities characterized by homogeneity—will be characterized by bridging forms of social capital. Participation in bridging communities provides access to a greater diversity of resources and thus, to a larger extent, contributes to combatting social inequality. Following this we can also expect that homogeneous communities will be characterized by bonding social capital, and more often contribute to the reproduction of social inequality rather than to social mobility.

By studying different forms of community—and the social networks that characterize these—we can understand better how social capital is built, reproduced, and activated. It is thus especially interesting to analyze different forms of community and their composition in order to understand the mechanisms involved in the formation of social capital.

Organizations are places where communities are built [26]. Organized and informal networks are both forms of social capital, and are often a result of individuals’ and groups’ strategies for gaining access to different arenas, or for strengthening their position in society. Participation in social networks can take place through formal organizational memberships, but also in a range of informal arenas. Research has shown that it is important that organizations are visible and that they are actively encouraging members to network, participate, and contribute [4]. In this article we analyze how organizations facilitate the building of networks, and which features of organizations facilitate the building of bridging and inclusive communities. Drop-in kindergartens are organizations that have as their specific purpose to form bridging communities. It is thus especially interesting to analyze the organizational mechanisms or conditions that facilitate the building of bridging social capital in these kindergartens, and focus on how different organizational conditions form the basis for the formation of different forms of community.

### 2.2. Organizations and the Building of Inclusive Communities

We focus here on organizations as a context for the building of social capital. In order to create open meeting spaces that promote participation and inclusion in the larger society, activities have to be organized in such a way that democratic processes are possible.

Morse pointed to how public organizations can promote participation and integration by enabling meetings between systems and citizens [27]. Creating a common mentality and a wish among the participants to contribute to the community is also absolutely necessary for an organization to become inclusive (Follett, in Morse) [27]. However, organizations provide different conditions for participation, and these partly depend on participants’ prerequisites and motivations. Building an organizational structure for participation such as an open meeting space, which attracts participants who do not have the motivation for, or the intention of, democratic participation is doomed to fail [27]. Organizations should therefore, in their work to encourage integration and participation, facilitate dialogue among their participants. Dialogue was seen by Follett as central to developing integrated groups, especially when groups comprise participants with a lot of different characteristics (Follett, in Morse) [27]. For organizations with ambitions to build bridging forms of social capital across cultural and social divisions, it is especially important to facilitate dialogue between members. Regular face-to-face meetings are important in the socialization of participants into an organization, as well as providing opportunities for groups to mobilize and participate vis a vis governmental agencies in organized arenas (Follett, in Morse) [27] (p. 7).

Research on organizations has been concerned with generating communities [26]. Wenger looked at organizational processes and mechanisms that facilitate the building of “communities of practice”—communities that are created inside organizations among colleagues [26]. Wenger was mainly concerned with how work communities are built and maintained, but similar mechanisms can explain how communities are built in other organizational contexts and between users [26].

Work communities and user communities are both expressions of what Wenger described as learning communities. Wenger put an emphasis on understanding how learning communities are created, and described organizational learning as maintaining and developing communities of practice [26] (p. 8). Three mechanisms characterize communities of practice, and are particularly important for creating a common mentality inside communities that are heterogeneous. Communities of practice are, first and foremost, built as a result of engaged participants who participate in activities that gather and maintain a community. It is thus important to have mutual participation among participants. Inclusion is key in order for participants to be able to engage and partake in a community, and mutual participation and engagement in a community leads to participants experiencing a sense of belonging in the community [26] (p. 74). It thus takes work to create a community of practice that is based on inclusion and participation, especially when a group consists of individuals with different but complementary characteristics and skills (communities that Wenger referred to as “complementary communities”). Wenger described communities as consisting of people who have similar and overlapping characteristics and skills as “overlapping communities.” In both of these forms of communities—overlapping (homogeneous) and complementary (heterogenous) communities—building community through mutual participation is important, as this is what “connects participants in ways that can become deeper than more abstract similarities in terms of personal features or social categories” [26] (p. 76).

The other mechanism that is important is negotiations between participants over common activities, and commitments to the community that are made visible through participation in these activities. Connecting around common activities is thus essential for building community. When participants share a common commitment to the activities and routines and a common understanding of the purpose of interaction, this facilitates the development of a sense of belonging and mutual commitments to the community they partake in (Wenger referred to this as “accountability”). When studying organizations, such as kindergartens, it thus becomes important to look at the activities of the organization and the ways in which these contribute to building and maintaining different forms of community.

The third mechanism that is key to developing inclusive communities is that the group develops a “shared repertoire.” A community develops routines, styles, language, and symbols that manifest the group’s practice and make visible the identity of the group [26] (p. 83). Wenger argued that developing a shared repertoire is key to inclusion as it is through shared repertoires that we “become invested in what we do as well as in each other and our shared history. Our identities become anchored in each other and what we do together” [26] (p. 89).

Boundaries develop within communities of practices—boundaries that both include and exclude [26]. Idiosyncratic ways of interacting develop in communities, and this often makes it difficult for outsiders to participate and be invited in. Similarly, understandings, language, and symbols that can be alienating and excluding can develop, and the group’s repertoire can seem self-referential rather than inclusive by making it difficult for new members to partake in a culture that is understood, but often not articulated [26] (p. 113). A repertoire in a kindergarten could be understood by looking at the organized activities in the kindergarten—and especially the language used to describe these activities, and the cultural content of the activities—and it becomes important to analyze whether there is shared repertoire among users, and whether users experience a sense of belonging to the organization.

Skills are also developed in communities (something Wenger referred to as the “competence” of the community). In a community, some members are marginalized, either because they do not possess the skills (“competence”) that are important to participate fully, or they are marginalized because they have not been granted appropriate access to participate (“marginalities of experience”). Membership in communities is also often mediated by institutional conditions [26] (p. 169). Wenger pointed to the importance of understanding how organizational features (something Wenger referred to as “design”) facilitate the building of inclusive communities and enable full participation in a community. We should thus examine whether an organization’s way of working promotes communication, sharing of experiences, and participation. In order to build inclusive communities it is also important that all perspectives are heard and that some groups are not marginalized, or made invisible [26] (p. 247). Wenger also argued that because communities are based on learning, it is important that organizations develop activities that are shared, build a shared identity, and involve and activate participants in a way that builds not only networks, but also a sense of belonging to a larger community.

Wenger described the importance of identifying and analyzing the organizational conditions that facilitate the building of different forms of community. In this article, we build on Wenger’s understanding of various mechanisms that are a part of the building of organizationally generated communities, and we empirically investigate the mechanisms that facilitate bridging forms of communities.

### 2.3. Organizational Design and the Building of Networks

The theoretical basis for our analysis is Wenger’s understanding of organizational designs and their importance in the building of learning communities [26]. Organizational designs include different forms of organizing—or different features of organizations that encompass organization [26].

Several have written about the uniqueness of organizations that have as their purpose to build welfare or fulfill social tasks—organizations that have ideological and social goals [28,29,30]. Repstad describes analytical categories that can be a part of the organizational designs of welfare-producing organizations [29] (p. 132). These categories are (1) the organization’s purpose or goals, (2) the organization’s ideology or vision, (3) the organization’s way of working or technology, (4) the organization’s structure—formal and informal, (5) the organization’s resources —material, and in the form of personnel, and (6) the organization’s relationship to its environment. These categories form the basis for analyzing connections between the goals of the organization and organizational design, and the connection between organizational designs and the conditions for organizationally generated communities. Welfare-producing organizations often have as their goal to build learning communities, and several features of their organizational designs (the organization’s ideology, way of working, structure, resources, and relationship to its environment) impact the conditions for building communities inside the organization.

Vabø and Vabo demonstrate—as does Repstad—the differences in how businesses operating in a market, and welfare-producing organizations, define their goals [31]. The organizations that deliver services for the welfare state (welfare-producing organizations) have as their mandate to serve societal goals, and they have to relate to a range of political stakeholders. Businesses that operate in a market have to relate to a different set of goals and stakeholders. The kindergarten sector is characterized by a diversity of private and public kindergartens that all have to relate to the same governance frameworks, plans, and financing schemes. The kindergartens also have to take into account the relations with their owners, and the users they serve. Care-oriented organizations’ (and kindergartens’) goals are thus often ambivalent, diffuse, and filled with contradictions [29] (p. 138), and are to a large extent shaped by the actors in their environment, as well as by the welfare–political context they operate within [30,32]. Welfare-producing organizations with different ownership structures—such as kindergartens—are thus particularly interesting to study, as their values are tied to sociopolitical goals and ideals as well as to a range of stakeholders and users that often have contradictory and varied expectations of the services they deliver [28] (p. 320).

We highlight various organizational features—or organizational designs—of drop-in kindergartens and analyze how these impact the conditions for building various forms of communities and networks. We explore the connections between different ways of organizing, and organizations’ ability to pursue goals of building inclusive communities. We also explore to what extent different understandings of the purpose of the organization affect whether different kindergartens produce bonding of bridging forms of community.

## 3. Methods

We analyzed various data sources from six different drop-in kindergartens. Drop-in kindergartens differ from standard kindergartens. Children are not offer a fixed place, and even if many are strongly encouraged—and in some cases referred to drop-in kindergartens—attendance is voluntary. Drop-in kindergartens also offer services to adults, where parents and caregivers participate with their children, and their services are a part of a political welfare initiative directed at caregivers and children. Drop-in kindergartens are subjected to the same regulations as standard kindergartens, as stated in the law for kindergartens and the national framework plan [1]. This means that the users of these kindergartens are to partake in pedagogical activities and learning activities. The number of drop-in kindergartens in Norway is declining. Statistics from 2018 show that there are 117 drop-in kindergartens in Norway, 43 of which are public and 74 of which are private kindergartens [33]. Haugset et.al. reported that there were 222 of these kinds of kindergartens [34]. White Paper 24 2012–2013 *The Kindergarten of the Future* described how drop-in kindergartens are viewed by the government [35]. The White Paper stressed that drop-in kindergartens are mainly a low-threshold service for caregivers and children who are not taking part in standard kindergarten services, that drop-in kindergartens are intended as a recruitment arena for standard kindergartens, that they shall contribute to developing social networks for families with small children, and that they have as one of their functions to identify children with special needs—including children who need language training—early [34]. The welfare state thus set clear goals for drop-in kindergartens, and these include the building of social capital, inclusion, and the equalization of social inequality for families and children that are not partaking in—and receiving services from—standard kindergartens. The government’s goal is to enroll children in standard kindergarten programs, and municipalities are seeking to save costs. As a result, drop-in kindergartens are struggling for funding and do not have a secure standing in many municipalities.

This study is a case study where we compared the organizational features of six drop-in kindergartens. The units of analysis in our study are the various cases [36]. We relied on observations, interviews, and documents as data sources that allowed us to explore the cases. The data provided information on the importance, and usage, of the kindergarten from the users’ perspectives, on practices in the kindergartens, and on how the kindergartens organize to serve their users. The data thus allowed us to explore the research questions in the article on the importance, and building, of social capital.

The data were collected as a part of a national study of drop-in kindergartens that explored drop-in kindergartens’ functions, usage, and importance in Norway [34]. The analyses of these data resulted in a report that suggested that there are different user groups and various types of drop-in kindergartens. The analysis in this article further explored the organizational features of these kindergartens, and the relationship between organizational features and practices of inclusion in drop-in kindergartens. Using the concepts of bonding and bridging social capital or networks [4,6], we analyzed the data by focusing on understanding the organizational features of kindergartens—looking at how organizational features, and mechanisms inside these organizations, contribute to building different forms of communities and networks.

The sample of kindergartens allowed us to study a range of kindergartens (Table 1). The kindergartens varied by location, ownership, personnel resources/employees, and profile. We selected the kindergartens based on information from the BASIL 2013 register and municipal webpages. We also contacted collaborating organizations to map which cases would provide us with a range of various kinds of kindergartens in our sample.

The data on these kindergartens were composed of a range of sources. We collected document data, mainly planning documents, and analyzed these. We observed practices in these kindergartens using a participant observational approach and a structured observational guide. We observed the indoor and outdoor environment in each kindergarten, the pedagogical practices, interaction between employees and users, and interaction and networking practices between users in the kindergarten. Additionally, we interviewed the employees in the kindergartens. These interviews were semi-structured, and were transcribed and later analyzed using a stepwise inductive–deductive approach [37].

The users of the kindergartens were also interviewed. We used a structured interview guide, and the answers from users were recorded on the forms during the interviews. These interviews were conducted in conjunction with the observation, and users were recruited to be interviewed during the fieldwork. In kindergartens with users that had Norwegian as their second language we used an interpreter during interviews and fieldwork. In these kindergartens the employees also helped identify and recruit users to ensure that we interviewed representatives from minority groups. The employees also helped with finding a space where we could conduct the interviews inside the kindergartens, and offered to look after the children when parents and caregivers were interviewed. Some of the interviews with users were conducted in common areas, while the parents were looking after the children. This could have affected the interviews, as various users had different amounts of attention and focus to devote to the interview. We interviewed a total of 10 employees and 36 users in the six cases. The data from the cases were anonymized and we refer to the kindergartens as case A, B, C, D, E, and F.

The analysis made visible a relationship between the organizational features of the kindergartens and how the kindergartens function as arenas of inclusion for users. We also observed that various user groups represented various forms of communities. The communities we observed in the kindergartens were different in several respects. Some user groups were composed of participants with similar social and ethnic backgrounds, whereas others were more diverse. These findings encouraged us to look at various user communities and the mechanisms that are involved in shaping and building these communities. What could explain the variations in the kinds of communities we observed in the various kindergartens? How does the organizational context and features of the organizations affect the composition and characteristics of the various user communities we observed?

Our analysis was inductive, moving from empirical observations to analysis of relationships and connections among the data. The categories in our analysis were generated from the empirical data, and thus reflect various aspects of organizational designs. We were particularly interested in the importance of organizational designs [26], and in how organizational features affect the building and consolidation of communities. The analysis was also abductive (stepwise inductive–deductive approach) in that we discuss our findings in relation to categories and mechanisms identified and defined in previous organizational studies and research focusing on organizational designs [26,29,37].

## 4. Analysis

In our analysis, we highlight organizational features that are of particular importance for building communities in kindergartens. We pay particular attention to two overarching features of kindergartens: (1) their purpose/goals and strategy, and (2) their structure—the way they are organized – including the content of their activities, their users, and their relationship to the environment surrounding the organization.

As a part of the organizations’ goals and strategy we look at the importance of various forms of ownership, as the owners of kindergartens affect their goals and purpose. We also look at the kindergartens’ profiles. The kindergartens have different target users, and this becomes visible in the kindergartens’ practices of profiling and marketing their services. Drop-in kindergartens’ profiles often reflect sociopolitical ambitions of creating open arenas for networking, and ambitions of meeting political welfare goals to integrate and include marginalized users. Structural features of the kindergartens include their localization—where they are located, whether the kindergarten is collaborating—and co-producing services—with other services, as well as the physical and material resources the kindergarten possesses as a result of various forms of organizing. We also look at the pedagogical focus and content—that is, the various curricular activities the kindergarten offers, and how the kindergarten organizes learning activities for its users. The last feature we look at and compare across kindergartens is the variation in the organizations’ participants—this includes the employees and the composition of user groups in the six kindergartens.

We discuss how various organizational designs—the organization’s goals and strategy (ownership and profile) and the organization’s structure (localization, pedagogical focus and content, and participants) affect the kindergarten’s ability to—and ambition for—building social capital. Our analysis shows that some features of kindergartens are integral to building inclusive communities and bridging forms of social capital —the kindergarten’s learning activities and the composition of the user groups—and we pay particular attention to these. We also discuss the extent to which these kindergartens build inclusive arenas for networking, and the ways in which various organizational designs facilitate the building of bonding and bridging forms of social capital.

### 4.1. Organizational Goals and Strategy

#### 4.1.1. Ownership and Goals

The first organizational feature that is of importance is the ownership of the kindergarten, and the relationship of the kindergarten to its owners. The kindergartens’ owners impact in a direct way the goals and purposes of the kindergartens, and thus also the kindergartens’ pedagogical activities and appeal to various user groups.

Three of the kindergartens in our sample were subdivisions of private or ideal/religious organizations (cases A, B, and C). They supported and served the owners’ overarching goals. The owners represented in our sample were one voluntary organization, a museum, and a religious denomination (cases A, B, and C, respectively). The other kindergartens were operated by municipalities. The role of kindergartens vis a vis their owners is to convey values that support the owners’ vision. Kindergartens represent one of several offerings the owner has as a part of their operation, and kindergartens are often co-located with the ownership organization. In one of the cases the kindergarten played a crucial role in financing the owners’ main activities as an ideal organization (case A)—the kindergarten financed the salaries of one employee who ran the kindergarten, and who at the same time contributed to running the organization’s daily activities. The pedagogical leader in this kindergarten described the tight coupling between the owner’s values and goals and the goals and content of the activities of the kindergarten:

*We want the kindergarten to focus on farming, nature experiences, physical activity, and community between children and adults. Through play, learning, and interaction we want the children to have good and exciting experiences, and to get a feel for how it was like to run a farm “in the old days.”*

The other kindergarten’s goal was also closely linked to the owner’s strategy and mandate to communicate particular cultural values. Additionally, the kindergarten was supposed to recruit users to the ownership organization, which was the local museum (case B). The pedagogical leader in the kindergarten explained that her hope was that children and parents who have visited the kindergarten will acquire historical knowledge and a sense of belonging:

*(We want them to) to have a good feeling when they later on step into an old house. These old houses do something with your senses, different rooms provide different feelings, an old room with dim lights like this one gives you different associations than being in a gym.*

One of the goals of the kindergarten was to teach, and maintain, knowledge on culture and conservation. The third kindergarten was also run by an ideal organization, and was co-located with a religious denomination. In this kindergarten the owners’ values formed the basis of the daily activities of the kindergarten (case C).

Facilitating the prevention of marginalization and the building of networks were main goals of the other kindergartens in our sample (cases D, E, and F). These kindergartens were all publicly owned—their owners were municipalities—and had a stated goal of integrating and preventing marginalization of vulnerable populations. They had as their goal to be an arena where parents and children who do not participate in the standard kindergarten services can meet. One of the pedagogical leaders explained that the owner’s purpose was to build an arena that prevents loneliness among parents and children:

*The fact that you get up in the morning, and fix yourself, eat breakfast, get out of the house, and do not sit at home in your sweatpants all day long, with the remote control in your hand. You are outside and creating good experiences with your children.*

The employees in these kindergartens stressed that an important part of their purpose is to provide a service to adults, and to be a gatekeeper for minorities and marginalized users that can provide access to the local community and the larger society:

*If you get to a municipality and do not know anyone, then you can come here and get to know someone. That in itself is preventive, to have someone to say hi to when you meet them at the store, to not get lonely, it is a way to get a foot in the door when you move to a new place … (Coming here) can be the first meeting with Norwegian society for some parents, apart from coming here they only spend time with their close family (pedagogical leader, case D)*

The last kindergarten in our sample (case F) was also owned by a municipality, and had a larger amount of non-ethnic Norwegian users. This kindergarten, as well as the other two municipally owned kindergartens (cases D and E), all had as their goal to include marginalized groups by providing opportunities to participate in common activities and meals. One of the kindergartens (case C) also offered warm meals to its users, and was very conscious of its goal to integrate and include its users in a community. This kindergarten was a privately run organization, but shared the same goals as the kindergartens operated by the municipalities—to work to prevent marginalization.

We saw that the kindergartens in the sample were working to achieve the same goals as their owners, and that they were actively being used to promote the goals of their owners. Some of the kindergartens played a key role in promoting the profile of their owners (case A and case B). The curricular content in the kindergarten was closely linked to the services that the owner provided for their customers—members and users of their services outside the kindergarten—and the kindergartens functioned as an extension of the owners’ operations—which did not necessary have similar goals of inclusion and the building of communities. Other kindergartens had as their primary goals inclusion and the building of community (cases C, D, E, and F). The owners started these kindergartens with the goal of becoming arenas that include and work as gatekeepers to the larger society. These kindergartens had as their stated purpose to facilitate the building of bridging networks and communities.

An interesting question is the extent to which these drop-in kindergartens accomplish their goals of building inclusive and open communities of the kind that Wenger described [26]. We saw that the kindergartens had different overarching goals that influenced and steered which kinds of communities could be built, and that whether they were successful in building bridging forms of communities depended on the ways in which they organized to reach these goals, and on how they collaborated and interacted with the environment surrounding the kindergartens.

#### 4.1.2. Profiles

Profiles show how organizations are seeking to represent themselves to their environment and to their users, and how they are working to make visible their identity. Most of the kindergartens introduced themselves on the webpages of their respective owners, and some also did their own marketing. Planning documents also revealed part of the work involved with making clear the profiles of the kindergartens. In the kindergartens that were closely connected to their owners’ profiles (case A and case B) we saw a clear focus on marketing, and on communicating how the kindergarten and its activities supported the goals of the owners. The first kindergarten was clear that it was building, and marketing, a unique brand (case A). The owner explained that she was conscious that she had to offer activities that draw users to the kindergarten, and that they actively promote the activities that they provide and that set them apart from others:“*Some have health providers on site, we have horses*.” The owner described a competitive situation in the market that makes it necessary to be clear when marketing services to users, and to be conscious of who their competitors are. They competed with several other kindergartens, but also with other private providers of activities. One of their competitors was the city’s swimming center. The leader in the other kindergarten who also marketed their services (case B) explained that they had not been successful in collaborating with the municipal refugee center, as the kindergarten’s profile attracted a certain kind of user:

*They (the refugees) came one time and sat outside here feeling cold. I think the users that come here more regularly seek the romantic notion of farming, and they use us because they like the unique atmosphere we have created here.*

Both of these kindergartens (case A and case B) were marketing their activities, which provided access to a certain set of experiences. By being clear that they were doing so, they were in a way promoting a “hidden curricula” that appeals more to certain kinds of users. Doing this kind of selective marketing of activities and experiences makes the services more meaningful and relevant to some users, but the kindergartens also contribute to a form of selective selection and recruitment of users. The effect of such profiling/marketing is thus that the kindergarten attracts a more homogeneous group of users. For example, one of the kindergartens that collaborated with the local museum (case B) attracted a certain kind of user who is interested in, and values, Norwegian culture and heritage. One consequence is that the users of the kindergarten are alike in many ways—they have a higher education and share an interest in culture—and as a result, the kindergarten’s work to establish and promote a profile leads to exclusionary rather than inclusionary practices. By focusing on certain segments in the market the kindergarten can facilitate the building of bonding forms of networks and homogeneous communities (overlapping communities, as in Wenger [26]), which often hinder rather than build bridging forms of network and social capital.

The kindergartens that marketed themselves as inclusive social arenas (cases C, D, E, and F) emphasized language, common activities, and the inclusion of a wide range of cultural diversity. One of the kindergartens stressed on their webpage that everyone shares the responsibility for creating a space in which all users feel welcome and a sense of belonging. They often had common activities that were arranged with this in mind, that took language barriers into account, and that focused on connecting people with each other, bearing in mind that many of the users had few networks outside the kindergarten. These kindergartens also displayed on their webpages the partners from the municipality whom they collaborated with, and they actively promoted them in their operations. Collaborative partners often included municipal health services (nurses, child protective services, family services, dental services). By doing this kind of profiling the kindergartens signaled that inclusion and the building of communities are integral activities, and that access to—and collaboration with—preventive social services are key to building inclusive communities. At the same time, this kind of profiling may have given parents and caregivers who did not receive, or were in need of, these kinds of services (e.g., language training, health and social services) the impression that the kindergarten provided services for marginalized groups only. As a result, the kindergarten’s profiling strategies could also work to exclude some users and stereotype the kindergarten as a place more suitable for a particular kind of user.

### 4.2. The Structure of the Organization

#### 4.2.1. Localization 

The localization of the kindergarten also affects the ways in which communities and networks are built and maintained. The localization of the kindergarten includes where the kindergarten is physically located, as well as material factors such as the use of space and indoor and outdoor facilities.

Localization is primarily about location and accessibility. Three of the kindergartens were located in the center of villages or smaller cities (cases A, D, and E). Accessibility affects the possibilities for the recruitment of users to the kindergarten. Many of the minority families did not have access to a car—they depended on getting a ride from someone, or on public transportation—which was described by many as a barrier for participation. Several of the users emphasized that it was important that the kindergarten be located close to where they live.

A close connection to the local neighborhood and low mobility among users also contributes to bringing together users from the local community—often this also implies users from similar ethnic and socioeconomic backgrounds. We especially saw this in one of the kindergartens (case C) where the majority of the users were non-ethnic Norwegians, and mainly representative of one ethnic community. The leader of the kindergarten explained that localization was the main contributing factor to the user group being so homogeneous: *“They (the ethnic community) live around here… and we have established ourselves in a community with them (the minority group).”* The kindergarten had not chosen to market their services to this particular ethnic group, but the location of the kindergarten, and the composition of the surrounding neighborhood, together contributed to creating a homogenous user group. The users in this kindergarten shared a language and a repertoire, but this was not due to the learning activities the kindergarten provided (as Wenger 1998 [26] pointed to). The kindergarten thus became an arena where bonding networks are built, and an arena where networks that were already established in the neighborhood are maintained. This was happening despite the fact that the kindergarten (case C) expressed that they wished to build connections between minorities and the majority in the area—and that they wished to work to include this minority into the larger society. Localization thus affects both the composition of user groups, and the conditions that facilitate or hinder the production of bridging forms of social capital.

Whether the kindergarten was co-located with another operation was also of importance. Five out of the six kindergartens in our sample shared facilities with another business or operation (cases B, C, D, E, and F). Three of the kindergartens shared facilities with other municipal services (cases D, E and F). The municipality had chosen to place the kindergarten with these other services in order to better serve their users, and to link various welfare and preventive services (such as health services or social services) together into a convenient and coherent offering. The purpose of co-locating these services was also to facilitate the referral, and introduction, of users to preventive services offered by the municipality or other collaborating organizations. By choosing to locate the services in the same buildings or in coinciding buildings, the kindergarten facilitated the building of linking forms of capital that provide users with access to various kinds of welfare services. One of the employees explained that the kindergarten had an explicit goal of offering preventive services:

*The threshold is supposed to be low for, e.g., introducing someone to child protective services. And if someone is struggling with psychological issues, then we may say, ”You know, I have a colleague down the hall, do you want to talk to her for a bit?” rather than saying that they need to set up an appointment and come back later.*

Two of the kindergartens (cases A and B) were also co-located and shared facilities with their owners. However, in these cases it was more obvious that it was access to material resources—such as playgrounds and indoor facilities, and access to animals and other outdoor facilities—rather than ambitions to build linking forms of capital that was the rationale for the co-location.

Localization is also about material resources—such as the furnishings of the rooms and access to materials for employees and users. Material resources also facilitate the building of networks in the kindergartens. Having a common area makes it easier to initiate networking, and also provides users with a place to gather. We saw from our observations that kindergartens that lacked common space, and that had a lot of restrictions in how they could utilize the space they had, had a harder time facilitating networking through common activities. Two of the kindergartens in the sample were located in facilities that were originally built to host kindergartens. In these kindergartens they had rooms that facilitated playing on the floor, and provided parents with the ability to participate in children’s play and thus also to engage in these activities with other adults. The other kindergartens were in facilities that were not designed for kindergartens. The facilities were often noisy, had bad sound quality, and lacked access to outdoor facilities. One of the employees (in case C) explained that the kindergarten’s facilities affected the possibilities for creating spaces for interaction: *“The room carries the sound in a bad way, it has poor acoustics and it quickly gets noisy here. When the mothers sit and talk this creates a lot of noise and it disturbs the children’s play.”* The noise in the room implied that people had to sit really close to each other in order to communicate. We saw that users in this kindergarten tended to not sit down, or that they were often sitting in segregated groups where they were primarily speaking their first language, as opposed to Norwegian. The facilities were also mentioned, as they were important for encouraging parents’ participation in and quality interactions with children: “*We have chosen to come here because it is important to get out of the house and not only sit at home, but also because they have such nice facilities for outdoor play for the children*” *(mother, case D)*. Access to material resources was mentioned by several of the users as one important reason for them using the kindergarten.

In summary, we saw that the kindergartens’ location and physical space impacted which kinds of communities were built in the kindergarten. Various aspects of the space kindergartens had available facilitated or hindered the ability to gather around common activities. The space available thus, to varying degrees, facilitated the building of networks. The facilities and access to material resources could also affect which kinds of users were motivated to use the kindergarten and the composition of the user group, and thus the very conditions underlying the ability for social interaction and the creation of networks.

#### 4.2.2. Pedagogical Focus and Content 

All the employees in the kindergartens stressed the importance of pedagogical activities in the building of community, and that the pedagogical practice has to be adjusted to the unique context and to the kinds of users the kindergarten has. The kindergartens were expected—clearly stated in laws and regulations—to be pedagogical institutions, and the employees used pedagogical work actively to build communities. One employee in a kindergarten (case D) explained that the pedagogical activities were chosen to facilitate bringing users together:

*We work actively to create networks between the parents. Concretely, we do craft activities, and the parents get very engaged, and if we create activities that make it necessary to talk together, then they get to know each other quickly and we create bonds as they do stuff together.*

The pedagogical curricula was important, as it provided the participants with something to gather around. Several of the parents in the various user groups often did not have anything else in common besides the fact that they met in the kindergarten—and that they had children that were the same age—and because of this, gathering around a common activity became especially important. Common activities could be crafts, reading books, arranging costume parties, or going on trips. The pedagogical focus of activities also affected which users were drawn to the kindergarten. The fact that kindergartens promoted the pedagogical nature of their activities helped attract users that sought to get access to learning activities—users act like consumers (e.g., cases A and B). Interview data also showed that many parents stressed the importance of mentoring and support for parents as another reason why they sought out the services of the kindergarten (cases D, E, and F).

There are a range of pedagogical resources that can be used to encourage participation in common activities. By using, for example, didactic models that emphasized prerequisites and goals for participation in learning- and experience-based activities, the employees worked to find activities that made it possible for everyone to participate. We also saw that employees wanted parents to engage and participate in the implementation and execution of learning activities, and that the kindergartens’ services in this way were strengthened through the resources the parents brought to the kindergarten. Kindergartens that had more heterogeneous user groups seemed to focus more on developing activities that facilitated networking between parents. Parents in these user groups often had nothing in common apart from meeting in the kindergarten, and having children that were the same age. The pedagogical activity thus became the focus of the interaction, and the basis for any networking. At the same time, it is challenging to get everyone to participate in common activities when the user group is heterogeneous. We did see, however, that getting everyone to participate was an easier task in kindergartens where one ethnic group did not dominate (and where one group’s repertoire did not dominate), and where the relationship between minority and majority was more balanced (cases D, E, and F).

We saw that the employees in the kindergartens sought to produce what Wenger described as communities of practice [26]. Gathering parents and children around common activities creates the possibility of building learning communities with a focus on mutual participation and a common understanding. This was done by singing songs that indicated a shared sense of community, by going on trips in the local neighborhood, or by making visible other identity- or place-based communities. Making the practice common included various forms of “working with” learning activities, and the kindergartens that succeeded with this chose carefully the activities they did in order to make sure they did not exclude minority users. We also saw that the kindergartens that succeeded with inclusion emphasized mutual commitments—that everyone was responsible for participating in, and shaping, the activities in the kindergarten. Over time, common routines, language, and symbols developed and came to constitute the repertoire of the kindergarten community. The kindergartens that succeeded with integration also focused on educating and teaching parents from minority backgrounds—or parents who were marginalized in other ways by not being integrated into educational institutions or the labor market—the skills they needed to initiate and participate in new networks. They worked actively to organize their activities to ensure that they were unifying, building a common identity, and engaging the participants—their resources and networks—in a way that created a sense of belonging to a larger community. By doing this, the kindergartens became one part in a greater effort to socialize and motivate individuals to be equal and full participants in society (as described by Follett, in Morse) [27]—and at the same time they ensured that users were acquiring the competence and networks needed to ease the municipalities work of integrating marginalized populations.

#### 4.2.3. Participants: Users and Employees 

Follett argued that having a common orientation towards building community is important for succeeding with integration and participation [27]. This implies that the participants in an organization—users and employees—have to be motivated to participate in common activities and active in building a community. Two aspects of users thus become important for the building of communities: their motivation for using the kindergartens’ services, as well as the composition of the user group and the resources the users bring into the kindergarten. In some of the kindergartens we found that users mainly represented the majority population in Norway (cases A and B). In case A and B the user groups were described as homogeneous and consisting of users from the majority population. Most users were ethnic Norwegians, and many were parents who were on maternity or paternity leave, or they were part-time workers. In case A some au pairs with Asian origins had used the services for a while, but the leader of the kindergarten argued, “*mainly parents that are on leave, or parents who work shifts use our services*”. In case B we found that partners of academic faculty had used the services of the kindergarten, and that faculty who needed a service for their children when they were working in Norway for a shorter time would use the services of the kindergarten. With the exception of one of the kindergartens (case C), the user groups in the other kindergartens were more heterogeneous (cases D, E, and F).

The user groups in the kindergartens had different motivations for using the kindergartens’ services. Characteristics of the users also affected how social capital was produced in the kindergartens. Several of the users had scarce social networks; they were not included in the labor market and they sought out the kindergartens’ services to meet others—they sought out a community for themselves and their children. This was especially true for newcomers and users with minority backgrounds. One example was one of the users who described his motivation for using the kindergarten’s services:

*I do not have an extensive network in this city, my network is small. I find that people are skeptical towards strangers and foreigners. They are reserved. So coming here is a social thing, for me.*

This user is an example of what we refer to as a “community-oriented user.” Other users had many networks outside the kindergarten. These were often parents who had consciously chosen to delay enrolling the children into standard kindergarten because they wished to be at home with the children—they sought out the kindergarten in order to meet their friends and acquaintances and to have a place to play with their children. One user, who was ethnically Norwegian and chose to be at home with the children, described her motivation to use the kindergarten’s services:

*I have gotten to know others in the kindergarten, but we only see each other when we are there, never outside the kindergarten. I have a large network in the local community, and additionally I have a friend who is also at home with the children and whom I spend a lot of time with.*

Additionally, we saw that some parents who did not yet have standard kindergarten services used the services of drop-in kindergartens. The user group thus often became heterogeneous, and users had very different motivations—and different needs— for participating in the kindergarten community.

In one of the kindergartens (case B) the user group was mostly composed of ethnic Norwegian parents who were on parental leave. The number of fathers in the user group also increased as the governmental policies related to parental leave for fathers expanded. We interviewed a mother in this kindergarten who worked part time and wanted to use her day off with her three-year old child. She explained that using her day off to visit the kindergarten allowed her to experience something with her child, and to provide her child with learning activities that they did not have access to in standard kindergarten services. At the same time she stressed that she could have chosen to use other services to create these shared experiences, or visited the child’s grandmother, instead of using the kindergarten’s services. She is an example of what we refer to as a “content-oriented user.” A content-oriented user is mainly concerned with the content of the learning activities rather than with building new networks.

A prerequisite for building bridging social capital is a community orientation and a heterogeneous user group (Follett, in Morse) [27]. Two of the kindergartens seemed particularly successful at creating a social arena where individuals from minority groups for whom Norwegian was their second language, and users who were ethnically Norwegian, met (cases D and E). Several of the users in these kindergartens argued that the cultural diversity was one reason why they were participating in the kindergartens’ services. One of the users in one of the kindergartens (case D) explained why she had chosen to participate in the kindergarten’s services during her parental leave, and argued that she saw the experiences she gained from participating as resources:

*The most important thing for me is that this is a nice and informal place to meet people. It is easy to get to know other children and adults here, and coming here makes my days more interesting. Even if I have an education I experience this kindergarten as a security net. The leader makes me feel safe and other parents also give a lot of good advice. I have also gotten to know a lot of people whom I would not have met. I have, for example, befriended a family from Afghanistan.*

A grandmother who visited one of the other kindergartens (case E) also described the cultural diversity as one of the main reasons why she took her grandchild to the kindergarten. The kindergarten was a social arena where one could meet people with other characteristics and backgrounds, something that was seen as valuable and important. She also stressed the importance of the facilities, that the user group was continuous, and that both of these factors also contributed to good and varied learning activities in the kindergarten:

*Here we have 25 nationalities represented in the kindergarten, and I see that for the mothers from Somalia this is an important community where women connect. I have gotten to know the regular users here. And these are people I would never have met if I did not come here.*

The examples above demonstrate a community orientation among these users. This kind of orientation was also stressed by the employees in the kindergartens who sought to be an open social arena. An employee in one of the kindergartens (case D), pointed to the importance of reciprocity between users and employees in the relationships created in the kindergarten:

*We must not forget that many of those who come here bring a lot of resources with them. They are eager to contribute, and they have something to contribute. It is not just us who give something here.*

It was mainly three of the kindergartens (cases D, E, and F) that succeeded in recruiting heterogeneous user groups. In the other kindergartens we saw that the user groups were much more homogenous. This was—as discussed earlier—a result of the localization of the kindergarten, and in some cases it was also a result of informal processes of self-recruitment to user groups. One employee in one of the kindergartens that had a heterogeneous user group (case F) explained how these processes unfolded, and that the kindergarten tried to adjust its learning activities to the composition of the user group, all with a goal of creating bridging kinds of communities:

*They (the users) have recruited each other, like, “I know a mom, can she also come?” So it works like the jungle telegraph. We do not think of this as a service only for users with Norwegian as a second language, but we adjust our activities to the users that come here. When users that have Norwegian as their second language come, we focus more on Norwegian language and culture, and we try to be a “bridge builder” into Norwegian society. But we provide a space for everybody here…*

Networks were also built into kindergartens with more homogenous user groups (cases A, B, and C). The users in these kindergartens valued the kindergarten as a social arena, one where they could meet people who were similar to themselves, and people who had the same language and background. One mother explained, *“There are so many people here that I share things with. I meet other Muslims and we have common values and a similar background.”* Others experienced that language barriers made communication difficult, and that “cliques” often formed since in these kindergartens people tended to mainly initiate contact with the people they already knew or had something in common with. This kind of informal behavior among users thus contributed to the fact that relations in the kindergarten were mainly bonding kinds of relations—many had as their main motivation to maintain networks they had already established and relations that were close knit, rather than developing new networks in the kindergarten. This kind of informal behavior also impacted the ability to build a shared repertoire in the kindergarten. The kindergarten—as an organization—was often not able to break these patterns as they did not want to reject or turn users away, so they tended to adapt to the user groups they had when they organized activities.

Despite the fact that employees in several of the kindergartens reported that most users were seeking to participate—they wished to participate in new networks and meet people who were different from themselves—we saw that this was also often not the case. Even if the personnel in the kindergarten have a goal of offering services that contribute to integration, this does not happen if the users do not have an interest in, or motivation to, partake in building complementary communities [26]. If the users do not participate in the mutual commitments that the organization seeks to establish (e.g., speak and learn Norwegian, participate in activities, build new networks), the organization is not able to realize the ambition of building an inclusive community. For example, we saw that several of the ethnic Norwegian users saw the kindergarten as a place for “others” if the percentage of minority users was high. One ethnic Norwegian mother described the reputation of one of the kindergartens with a heterogeneous user group as *“a place for foreigners who do not have anywhere else to go… and there are a lot of older children there.”* When these informal patterns develop potential users also create understandings or stereotypes of the kindergarten, which again serve to strengthen the self-recruitment of users who seek bonding kinds of communities. In kindergartens where these dynamics of self-recruitment were present—despite the fact that the kindergarten described itself as “open”— a closed arena developed, with limited possibilities for networking. We also saw that majority users, who most often were ethnic Norwegians, saw the kindergarten as a place where they could spend time with their young children. However, drop-in kindergartens also had as their ambition to provide activities for older children, in order give them access to programs that are similar to standard kindergarten services prior to them starting school, and thus making the transition into standard kindergarten services easier. As a result, there was a tension between the ambition that drop-in kindergartens had—to include minorities and older children—and how many majority users saw the kindergarten services—as an offering for the youngest children where the parents could also meet people similar to themselves.

The composition of the user group was crucial for the possibilities for building bridging relations. In sum, we saw that the users contributed to reproducing particular kinds of relations and networks. The participants did this through their practice, and by maintaining or segmenting understandings of the reputation of a kindergarten. When users, and the surrounding community, defined the kindergarten as a place for a certain kind or kinds of groups, they contributed to reinforcing stereotypes such as, “This is not a place for my child and me.” The fact that a lot of majority users also had a content- and customer-focused attitude to the kindergartens’ services, or that they mainly sought a community “of their own,” challenged the kindergartens’ ability to build bridging networks, even in kindergartens that had an explicit ambition of being open and inclusive.

Employees were also a part of the organization’s participants. Employees and their work conditions impacted how communities and networks were built. The resources the employees had to work with were of particular importance, and the ratio of employees to users affected the ability to build bridging social capital. One common feature of all these kindergartens was that they had few employees. Several of the employees described that they were extremely busy, and that they often felt unable to set aside enough time to work on building networks. Many users also meant that the everyday life in the kindergarten was hectic, and that it was difficult to arrange common pedagogical activities. Many also experienced that it was challenging to facilitate networking and community building when a lot of individual users needed their attention and advice.

The competence of the employees also affected whether they focused on networking and community building. All of the kindergartens were run by kindergarten teachers who had an education in childhood education. Some employees in these kindergartens did, however, combine their job with other jobs they held or were additionally doing other tasks for the owner’s organization. In three of the kindergartens (cases D, E, and F) employees also worked for other municipal health or social services, which eased the kindergartens’ collaboration with these services. The fact that employees had different backgrounds and tasks they filled did, however, affect whether their focus was on building social networks. Kindergarten teachers were mainly concerned with providing pedagogical activities that could help build community, and with using language training as a tool by which to do this, whereas those working with health and child protective services were more concerned with the users’ social and health challenges. We found that employees in the kindergartens that focused on integration and inclusion were, however, also focused on collaborating with other institutional services that could follow up with families that were in need of other welfare services. 

In sum, we saw that different organizational designs impacted whether the kindergartens built bridging forms of social capital (Table 2). Our analysis shows that certain organizational features of kindergartens are of particular importance for the building of social capital—the kindergarten’s profile, the kindergarten’s learning activities, and the composition of the user groups. These features also co-interact—it is, for example, not sufficient that the owner has a goal of inclusion and integration for the kindergarten (as in case C) if the composition of the user group is too homogenous. At the same time, the localization of the kindergarten—several of the kindergartens are located in the center of a smaller city—and co-location with other services make it easier to recruit heterogeneous user groups (cases D, E, and F). The fact that several of the kindergartens emphasized community-oriented activities, and marketed themselves in ways that attracted a more varied group of users, contributed to their success in producing bridging forms of networks between users.

## 5. Discussion

### Organizations and Social Capital 

We used Wenger’s approach to organizations [26], and found that the kindergartens developed different kinds of communities with different prerequisites for building new networks. We saw the contours of two different types of organizations. Some of the kindergartens had a clear profile, and their profile reflected the goals of their owner’s organization. These kindergartens we referred to as “profiling organizations” (cases A and B). In these kindergartens the marketing of services was influenced by the owner’s goals, and this affected which learning activities the kindergarten focused on, as well as the composition of their user groups. The user groups in these kindergartens were more homogenous and the communities that were built were primarily bonding—or overlapping—communities. Other kindergartens focused on inclusion, and we referred to these as “inclusionary organizations” (cases C, D, E, and F). The inclusionary organizations all had as their goal to be a gatekeeper to society and to facilitate networking. The employees in these kindergartens emphasized building bridging forms of community through learning activities that focus on mutual participation and shared repertoire. The user groups in these kindergartens were more heterogeneous, which was often more demanding to organize, but which also made possible the building of bridging (and complementary) communities.

The composition of the user group was the single most important factor affecting the learning activities in kindergartens and whether these activities contributed to building bonding or bridging forms of communities. If the user group was homogenous, common activities often contributed to strengthening the community and networks among users who were already in networks with each other, or who shared similar characteristics and backgrounds. In kindergartens where the user groups were heterogeneous, common activities contributed to a larger extent to the building of new networks, and to the building of bridging forms of social capital. When users’ motivation was to seek out new communities, this also affected their motivations to participate in new networks and to engage in the types of communities of practice that the kindergartens represented.

The users were key actors who shaped the communities and forms of capital that were built. A heterogenous user group is thus a prerequisite for building bridging forms of social capital. It is, however, important that users with resources and networks be willing to include others in their networks. We saw that parents that related to the kindergarten as customers shopping for different services and activities for their children. This led to a fluidity in their relationship with the kindergarten; they came and went and were interested in the experiences that the kindergarten could provide rather than in the community or network they participated in. The users’ motivation for using the kindergarten services thus also becomes an important prerequisite for building bridging forms of social capital. In kindergartens where strong bonding communities existed we saw a dynamic that often served to exclude those who were different, and that challenged the kindergarten’s ambition to be inclusive. “Unruly” users thus represented a challenge to the organization in reaching its goals.

Follett pointed to the importance of a “community-oriented attitude” in creating full participation and inclusion [27]. Several of the kindergartens in our study had users with a “content-oriented attitude”—they were often concerned with the content of the learning activities, and sought out a service that would benefit their own children. This attitude is also an expression of an individual focus that challenges the kindergarten’s effort to build community. Our analysis shows that the profiled organizations more often attracted content-oriented users, and that these kindergartens also focused on providing unique experiences and pedagogical content as a conscious strategy for recruiting users and for surviving in a competitive market. Kindergartens that were oriented towards content rather than community ended up recruiting more homogeneous user groups, as users with similar preferences were drawn to kindergartens with a particular profile.

This begs the question of whether more competition in the kindergarten sector contributes to an increasing segmenting of user groups. In combination with an increase in content-oriented users who mostly relate to the kindergarten as customers, this can lead to the fact that children with similar social backgrounds are grouped together in profiling organizations. If users to a greater extent relate to the kindergarten as a service provider, rather than as an arena for social interaction and community building, this can also challenge overarching political and social goals of kindergartens building inclusive communities. Recruiting users from neighborhoods where everyone has a similar social and cultural background leads to segregation of user groups and makes it difficult to build inclusive communities. Another question is whether segregation of user groups into different types of kindergartens with different services will challenge the goal of providing equal service offerings for all children.

Wenger pointed to the importance of a shared repertoire for building communities in organizations [26]. In the cases where the kindergarten recruited users with similar backgrounds, they already shared a repertoire prior to coming to the kindergarten (e.g., they spoke the same language or had similar cultural values). The kindergarten did not, in these cases, contribute to building new communities, but rather to maintaining and reinforcing networks that were built outside of the kindergarten. In kindergartens with heterogenous user groups—and where users had a community-oriented attitude —the focus was on building new networks and communities with a diverse, but shared, repertoire. The composition of the user group is thus critical to a kindergartens’ ability to be able to produce inclusive communities and bridging forms of social capital. Drop-in kindergartens provide a service that is organized according to the drop-in principle, which facilitates the creation of fluid communities. Some users drop by occasionally, whereas others are regulars. At the same time employees and many users want the kindergarten to stay in business, and they want to maintain the services, as it is a service and community they value.

Wenger stressed the need for mutual commitments as a prerequisite for building communities inside organizations [26]. We found that kindergartens varied in their ability to produce community-oriented practices. The users were important mediating actors; they participated actively in the production of various forms of community. At the same time learning activities were key to creating communities, and worked as a mediating mechanisms by contributing to a sense of belonging and fellowship between participants with various characteristics. The localization of the kindergarten—and the degree of co-localization with other services—also impacted whether the kindergarten had the prerequisites for building bridging forms of networks. Kindergartens that were co-located with other welfare services had more resources, which facilitated the building of bridging forms of networks. They often referred users to other services, and other services referred their users to the kindergarten, with the result that the kindergarten recruited more heterogenous user groups. Kindergartens that shared facilities with other services more often collaborated with these services on developing programs and activities. The users mediated these processes—they were co-producers who actively partook in the work of kindergartens—and users’ motivations affected the kindergartens’ ability to build bridging forms of social capital. In the kindergartens where user groups were heterogenous—and where the users had a community-oriented attitude—bridging networks were built. In kindergartens where user groups were homogenous —or where a heterogenous user group existed but their motivation was mainly content- and consumer oriented—bonding networks were maintained, and few new networks were built. Communities in these kindergartens—despite the fact that they had an ambition to be inclusive—were experienced by users as divided and exclusionary.

We saw that the bonding networks were not diverse—that most of their participants were individuals with shared characteristics—and that as a result, they did not contribute to the building of the kinds of inclusionary communities that many kindergartens seek to build. Bonding networks did build communities, but these were often non-inclusive, and they often did not provide participants with access to increased resources for mobility. Bonding networks thus often ended up building and reinforcing (often marginalized) subcultures rather than inclusionary communities. For groups that are already marginalized, these subcultures can be more destructive than helpful, as they do not help build trust between groups, or trust to other welfare-providing institutions. One could, of course, argue that bonding networks are a prerequisite for bridging networks, and that the relationship between bonding and bridging networks could be explored further. Our purpose in this article, however, was to look at whether the communities that exist in the different kindergartens resemble bonding or bridging forms of community. Whether the development of bonding forms of community in kindergartens can contribute to participants partaking in bridging forms of networks outside the kindergarten —and thus to integration on a societal level—is an important question, but not one we have the appropriate kinds of data to answer in this study. 

We did, however, see that in order to build inclusive communities of practice, the organization has to focus on building bridging forms of networks, and at the same time the users have to participate and activate the social capital they get access to in kindergartens. Our analysis has shown that several organizational features of kindergartens contribute to the building of social capital, but that the building of inclusionary and bridging forms of networks require that participants participate in practice-based networks where participants partake in common—and learning-based—activities.

## 6. Conclusions 

In this article, we use drop-in kindergartens as a prism for studying how organizations build bridging forms of social networks. Drop-in kindergartens are one of many organizations—or organized services—that have as their purpose to promote social inclusion and to be an open social arena—accessible to all. These kinds of initiatives are encouraged by the government and are seen as important tools that can be used to promote voluntary activity, social integration, and the building of social networks that contribute to the common good [38]. Our findings reinforce the idea that drop-in kindergartens are important institutions that both promote and build social capital, and serve to include and integrate marginalized populations into Norwegian society.

We found that kindergartens varied according to how successful they were at creating community-building arenas. In some of the kindergartens we saw that bonding forms of social capital were reproduced, whereas in others they focused on facilitating the building of new communities and bridging forms of social capital. Bridging forms of networks have a larger capacity to promote social mobility and provide access to other forms of capital. As we saw from our analysis, some features of the kindergartens were of particular importance for the building of bridging social capital—the kindergartens’ pedagogical focus and common learning activities, and the composition of their user groups. These features we understood as key formal features of an organization’s design. We also discussed how these features co-interacted with other organizational features to create different dynamics —or mechanisms—that explain how bonding and bridging forms of capital are built in drop-in kindergartens. More research on how various organizations facilitate networking and the building of bridging communities would provide a deeper understanding of social capital as a phenomenon and also provide us with a deeper understanding of which organizational conditions are necessary for building bridging forms of social capital in other welfare-producing organizations.

Our findings pointed to the relationship between the organization and its environment. Seeing organizations as open systems made us aware of the importance of users and their motivations. Networks are produced and reproduced in user groups, and we saw that informal processes among users play a crucial part. Users’ motivations for building community and partaking in institutional initiatives are also key. Organizations thus, to a large extent, depend on users’ motivations for, and attitudes towards, participation. Some users come to the social arena with a content- and consumer-oriented attitude, rather than a community-oriented attitude, and this attitude is reinforced in organizations that focus on particular market segments. Research should examine further how citizens interact with similar organizations and community-oriented initiatives, and how users’ motivations impact which kinds of networks are built. Research should also critically examine whether initiatives contribute to the leveling, or the reproduction, of inequality. Future research should also focus on understanding how organizations’ profile and motives impact their abilities to build social capital. We found that kindergartens that had a content-oriented profile were able to attract users, and thus had a competitive advantage on the market. At the same time, however, kindergartens with such profiles recruited more homogeneous user groups, and thus were often less successful at building bridging forms of networks. The question of whether the networks that are built in kindergartens extend and provide benefits beyond the immediate provision of service should also be explored further. Unfortunately, our data did not allow for this, although many of our informants claimed that they acquired access to extended networks through their kindergarten community. The importance of these networks in providing access to key networks with resources and power—to the labor market or to important arenas where decisions are made—should be explored further.

We argue that this study can inform studies of other organizations with similar goals to create open social arenas and facilitate the building of inclusive forms of community. One limitation of our study is our sample, which is small and representative of only one limited part of the public sector. We recommend that more studies be done in other welfare-producing organizations—both public and private—that have as their goal to build bridging forms of networks and social capital.

## Figures and Tables

**Table 1:** Overview of cases: presenting key features of the kindergartens.

Case	Geographic Location	Owner	Personnel Resources	Facilities	Interviews
Case A	Rural location, located in a mid-sized Norwegian city	Private: foundation linked to a non-profit organization	One part-time position for a preschool teacher and leader (40% position, and the daily manager contributes)	Farmhouse	2 employees (including owner and daily manager) 1 user
Case B	Large Norwegian city	Private: a part of a museum’s operations	One part-time position (adding up to a 60% position for a preschool teacher)	Farm connected to the museums area	1 employee 5 users
Case C	District in a large Norwegian city	Private: operated by a non-profit foundation based on religious values	Two part-time positions (assistant 20% and preschool teacher 40%, distributed into two various units)	Community building	2 employees 4 users
Case D	Mid-sized Norwegian city	Municipality	One full-time position (preschool teacher)	Former military building	1 employee 9 users
Case E	Small, rural city	Municipality	One part-time position (20% preschool teacher, school nurse contributes)	Community/family center	2 employees 8 users
Case F	Large Norwegian city, district with a high percentage of residents with minority backgrounds	Municipality	One full-time position (preschool teacher and daily manager 20%, and assistant 80% position)	Community/family center	2 employees 9 users

**Table 2:** Organizational features of drop-in kindergartens.

	Ownership	Profiling	Localization	Pedagogical Focus and Content	Participants
Case A	Private, voluntary, non-profit organization	Emphasis on the values of the non-profit organization, focus on recruitment to the organization.	Co-located with the organization, shared facilities, rural.	Knowledge of nature, agriculture, and animals. Common activities arranged where children and adults participate. Focus on experiences.	Predominantly ethnic Norwegian users, parents on maternity/paternity leave, majority of mothers, parents with connections to the labor market.
Case B	Private, museum	The kindergarten services are supported by and are supposed to support the museum’s activities. Focus on familiarizing children and parents with local history and traditions.	Co-located with a local historical museum, located in the center of one district in a larger city.	Knowledge of local history and local traditions. Playing and learning in a historical setting. Focus on experiences.	Predominantly ethnic Norwegian users, as well as some users with higher education from other countries affiliated with a university community.
Case C	Private, congregation	A social meeting place, focus on meals and socializing, also provide an evening-based service.	Co-located with a community house, placed in a neighborhood in a suburb of a larger city.	Provide meals and non-organized play time. Little focus on organized activities, ad hoc organized activities occur. Ambition of bridging the gap between minority groups and ethnic Norwegians, and integrating non-ethnic Norwegians into Norwegian society.	Homogenous user group, predominantly from one ethnic minority, users seek the services of the kindergarten for community building.
Case D	Municipality	A meeting place that caters to various users, the city in miniature	Co-located with a kindergarten, placed in the center of a smaller city	Provide non-organized play time and activities focused on language learning for both children and adults. Ambition to see and hear every user. Arrange activities for parents in addition to and outside of the kindergarten’s opening hours. Focus on parental mentoring and guidance.	Heterogeneous user group, a significant amount of users with minority background. Parents, grandparents, and users without connections to the labor market.
Case E	Municipality	Focus on prevention—preventive health and social services—and social inclusion.	Co-located with a health center, placed in the center of a smaller city.	Common and organized activities with an emphasis on language development and play. Focus on activities that require collaboration and interaction.	Heterogeneous user group, significant amount of users with minority background, several users are not connected to the labor market and are encouraged to participate in the services of the kindergarten to build networks.
Case F	Municipality	Focus on how to build competence among parents and on how to recruit users into other social arenas/familiarize users with other social services.	Co-located with a health center, placed in the center of a larger city.	Focus on non-organized play and music as a way to build language competence for users. Arrange theme days and lectures for parents on various topics related to children’s development and parental capacity. Focus on parental mentoring and guidance.	Diverse and heterogeneous user group, most of the users have minority background. Users have sought out the services of the kindergarten due to the content and multicultural nature of the services.

## Data Availability

The data presented in this study are available on request from the corresponding author.
